# COVID-19 contact tracing at work in Belgium - how tracers tweak guidelines for the better

**DOI:** 10.1186/s12889-023-16911-1

**Published:** 2023-11-03

**Authors:** Jerome Kieltyka, Jinane Ghattas, Sandrine Ruppol, Pablo Nicaise, Joren Raymenants, Niko Speybroeck

**Affiliations:** 1 CESI ASBL, Avenue Konrad Adenauer 8, 1200 Woluwe-Saint-Lambert, Belgium; 2 Institute of Health and Society (IRSS), Université Catholique de Louvain, Brussels, Belgium; 3 Laboratory of Clinical Microbiology, KU Leuven, Herestraat 49, 3000 Louvain, Belgium

**Keywords:** Contact Tracing, COVID-19, Workplace, Occupational health

## Abstract

**Background:**

When conducting COVID-19 contact tracing, pre-defined criteria allow differentiating high-risk contacts (HRC) from low-risk contacts (LRC). Our study aimed to evaluate whether contact tracers in Belgium followed these criteria in practice and whether their deviations improved the infection risk assessment.

**Method:**

We conducted a retrospective cohort study in Belgium, through an anonymous online survey, sent to 111,763 workers by email. First, we evaluated the concordance between the guideline-based classification of HRC or LRC and the tracer’s classification. We computed positive and negative agreements between both. Second, we used a multivariate Poisson regression to calculate the risk ratio (RR) of testing positive depending on the risk classification by the contact tracer and by the guideline-based risk classification.

**Results:**

For our first research question, we included 1105 participants. The positive agreement between the guideline-based classification in HRC or LRC and the tracer’s classification was 0.53 (95% CI 0.49–0.57) and the negative agreement 0.70 (95% CI: 0.67–0.72). The type of contact tracer (occupational doctors, internal tracer, general practitioner, other) did not significantly influence the results. For the second research question, we included 589 participants. The RR of testing positive after an HRC compared to an LRC was 3.10 (95% CI: 2.71–3.56) when classified by the contact tracer and 2.24 (95% CI: 1.94–2.60) when classified by the guideline-based criteria.

**Conclusion:**

Our study indicates that contact tracers did not apply pre-defined criteria for classifying high and low risk contacts. Risk stratification by contact tracers predicts who is at risk of infection better than guidelines only. This result indicates that a knowledgeable tracer can target testing better than a general guideline, asking for a debate on how to adapt the guidelines.

**Supplementary Information:**

The online version contains supplementary material available at 10.1186/s12889-023-16911-1.

## Background

Contact tracing is one of the non-pharmaceutical strategies allowing to prevent the circulation of severe acute respiratory syndrome coronavirus 2 (SARS-CoV- 2) responsible for COVID19, and other infectious diseases such as tuberculosis and monkeypox [1, 2]. Contact tracing consists of identifying people who have been in contact with an index case, infected by SARS-CoV-2 and who are therefore potentially infected, in order to enforce quarantine and testing. Although contact tracing has been extensively studied and is recognized as a method of preventing disease outbreaks, research on its effectiveness remains limited [3]. Moreover, most studies on contact tracing have relied on modelling and only a few reported findings from real-world implementation. Belgium’s public health authority used pre-defined criteria to classify contacts as “low risk contact” (LRC) or “close or high-risk contact” (HRC) [4]. Those criteria were based on the CDC’s definition of HRC and they have not been changed since the start of the pandemic. Testing and quarantine requirements have often differed according to risk classification.

In Belgium, occupational doctors were required by the authorities to assist with contact tracing. Regional units were responsible for contact tracing in the private sphere, while occupational health doctors were responsible for contact tracing in the professional environment [5]. The workplace has been identified as a source of potential infection [6].

Early in the pandemic, contact tracing guidelines had to be developed and implemented rapidly, with limited scientific foundation. This may have influenced the level of trust in the guidelines among healthcare workers and employees. Healthcare workers and other tracers, moreover often had no previous experience in contact tracing, and had to be trained on the spot. Both of these factors may have influenced the implementation of these contact tracing guidelines. While some data exist on the compliance of individuals subjected to testing, quarantine and isolation [7], the implementation of contact tracing itself, i.e.at the level of the contact tracer – requires further study.

The aim of this study is to investigate how contact tracing guidelines were implemented in practice by contact tracers with different profiles, through the following research questions:

- Compliance with guidelines: Are contact classification criteria, as defined by Belgian federal public health authorities, applied correctly by contact tracers with different backgrounds?
- The efficacy of the classification: Is an individual who has been classified as having a HRC more likely to test positive for SARS-CoV-2 than a person who has been classified as a LRC?
- The difference between theoretical classification and practical classification: Does the risk of infection of a HRC differ according to the type of classification, i.e. guideline-based or contact tracer-based?

## Methods

### Study design and context

This study was conducted via an anonymous online questionnaire on Qualtrics®. The hypertext link to the survey was sent by e-mail to a population of 111,763 workers whose employers were affiliated with CESI (Belgian external service for prevention) at the time of data collection, i.e. from September 21, 2021 to November 1, 2021.

### Questionnaire

The questionnaire was created jointly by several occupational doctors, a biostatistician and a public health doctor. It was then checked for coherence by two additional medical doctors and for intelligibility by twenty adults aged 28 to 66, working in various non-medical sectors (education, language, IT, real estate, etc.). A copy of the questionnaire is available in the appendix (Appendix 1).

### Statistical analysis

The database on Qualtrics was downloaded in the form of an Excel file and analyzed in Rstudio (RStudio 2022.02.3 + 492 "Prairie Trillium" Release).

We estimated the positive agreement (PA) and the negative agreement (NA) between classifications by contact tracers and the guideline-based classification [8] in order to determine whether the guidelines criteria had been followed by the contact tracer. We then stratified tracing according to the profile of the contact tracer.

We used the following variables to answer the first research question, i.e. the compliance with guidelines:

- The contact classification as defined by the contact tracer (HRC – LRC).
- The profile of the contact tracers: occupational doctor, internal tracer (internal prevention advisers, human resources, etc.), general practitioners (GP), other (which includes both “someone else” or “no one”).
- HRC or LRC, as per the public health authority guidelines. These variables were determined by applying a simple algorithm to answers to the questionnaire. If one of the criteria of a HRC was met, a contact was a HRC according to the guidelines-based classification. If none of the criteria was met, it was a LRC.

These variables allowed us to compare the guideline-based contact classification with the contact classifications by the tracer (Table 1). Both were self-reported by the index case.

**Table 1:** Summary of self-reported contact classification according to the guidelines and the contact tracer

Contact Tracer decision	Guidelines classification		
	HRC	LRC	total
HRC	a	b	a + b
LRC	c	d	c + d
Total	a + c	b + d	N

The formulas used for the PA and NA, were the following:

$$\mathrm{PA}=\frac{2\mathrm{a}}{2\mathrm{a}+\mathrm{b}+\mathrm{c}}$$

$$\mathrm{NA}=\frac{2\mathrm{d}}{2\mathrm{d}+\mathrm{b}+\mathrm{c}}$$

For the second research question, i.e. the comparison of infection risk between HRC and LRC, and third research question, i.e. the influence of the type of classification – guideline based or contact tracer-based – on the infection risk, we considered the following variables in order to determine which type of classification (by contact tracer or by guideline) was more efficient at identifying risk of infection:

- The contact classification by the contact tracer (HRC-LRC): independent variable.
-The contact classification as per the public health authority guidelines (HRC or LRC): independent variable.
- The result of the PCR-test carried out after the contact (Positive–Negative): dependent variable.

We performed a Poisson regression. The outcome was whether one tested positive for SARS-CoV-2 following the contact. Vaccination status was included as a covariate. Individuals who had received at least one vaccine dose a minimum of 14 days prior to contact and/or tested positive prior to contact were considered immune. The remainder were considered non-immune.

We calculated the risk ratio (RR) for the different variables. A *p*-value below 0.05 was considered statistically significant.

## Results

### Participants

Out of 111,763 workers invited by mail to participate in the study, 5525 provided at least a partial response and 4524 provided a full response. Of the latter, we excluded workers under the age of 18 and those who had not been in contact with a positive case of COVID-19 in the workplace. The remaining 2,422 workers were considered eligible for the study. Workers who did not answer at least 36% of the questionnaire were excluded, leading to 1105 patients being included for the first question, i.e. the compliance with guidelines.

**Fig.1:**
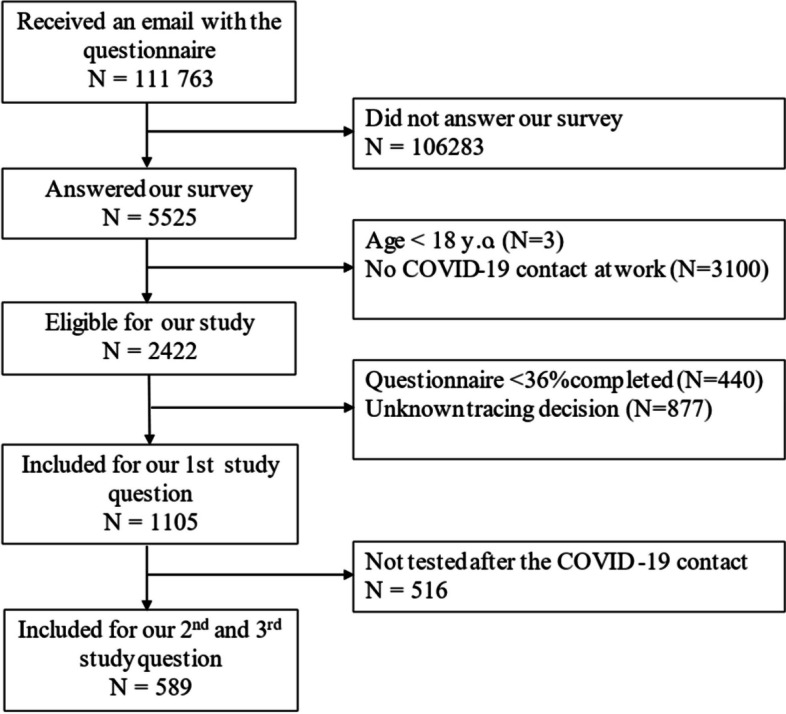
Flow chart of the selection of the study population, online survey on contact tracing efficacy, September 2021, Belgium

For the second research question, i.e. the comparison of infection risk between HRC and LRC, and the third research question, i.e. the influence of the type of classification – guideline based or contact tracer-based – on the infection risk, we excluded a further 516 workers who had not been tested following their contact, resulting in a final total of 589 participants, as presented in Fig. 1.

The demographic characteristics of the participants are presented in Table 2.

**Table 2:** Demographic characteristics of the population of respondents, the population selected for the first research question (population 1) and the population selected for the second and third research questions (population 2–3)

Variables	Number and proportion of individuals		number and proportion of individuals		number and proportion of individuals	
	All respondents		Population 1		Population 2–3	
Total	5163		1105		589	
Language						
French	5018	(97.2)	1079	(97.7)	572	(97.1)
Gender						
female	3362	(65.1)	764	(69.1)	415	(70.5)
Mean age	43.2 (18–70)		42.2 (18 -69)		42.0 (18–69)	
Education level						
none	16	(0,3)	1	(0.1)	1	(0.2)
Primary school	95	(1.8)	12	(1.1)	3	(0.5)
General secondary school	817	(15,8)	139	(12.6)	76	(12.9)
Professional secondary school	735	(14,2)	123	(11.1)	70	(11.9)
Higher education	3325	(64.4)	811	(73.4)	426	(72.3)
Working sector						
Education	573	(11.1)	124	(11.2)	53	(9.0)
Healthcare	2275	(44.1)	623	(56.4)	359	(61.0)
Social work	501	(9.7)	110	(10.0)	58	(9.9)
Other	1819	(35.2)	248	(22.4)	119	(20.2)

Our population of respondents consisted mainly of female (65.1%), French-speaking (97.2%) workers with higher education (64.40%) and working in the health care sector (44.1%).

We observed that the health care sector was overrepresented among participants compared to the general population.

There was a higher proportion of health care workers in the population of respondents than among the individuals we selected from this population of respondents for our three study questions. The other variables we investigated did not differ significantly between these populations.

### Positive percentage agreement and negative percentage agreement between contact classifications, stratified by tracer profile

The results of the first research question, i.e. the compliance with guidelines, are presented in Table 3. Out of the 1105 participants, 71 (6.42%) had been evaluated by an occupational doctor, 559 (50.59%) by an internal tracing team, 124 (11.22%) by their GP and 351 (31.76%) by someone else or no one (both included in “other”). We observe that contact tracers labelled the contacts as HRCs (rather than LRCs) more often than the official guidelines would have.

**Table 3:** Contact classification by contact tracers (CT) and official guidelines (OG)

	Occupational Doctor		Internal Tracer		General Practitioner		Other		Total	
	CT	OG	CT	OG	CT	OG	CT	OG	CT	OG
Total	71		559		124		351		1105	
Contact Classification										
HRC	31	24	247	174	83	65	144	101	505	364
LRC	40	47	312	385	41	59	207	250	600	741

Internal tracer = Any individual who has participated in contact tracing at work (prevention adviser, HR…)

*HRC* High risk contact

*LRC* Low risk contact

**Fig. 2:**
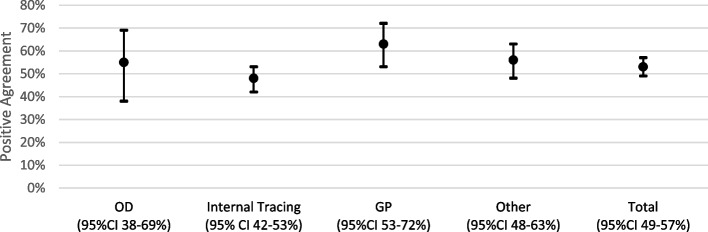
Interval plot of positive agreement of risk classification, by contact tracer profile

**Fig. 3:**
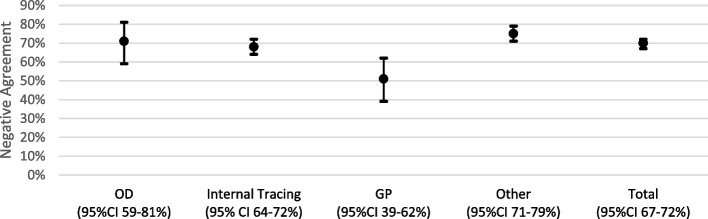
Interval plot of negative agreement of risk classification, by contact tracer profile

The positive and negative agreements of the guidelines-based and contact tracer-based classifications are presented in Fig. 2 and Fig. 3. Without considering the contact tracer’s profile, positive agreement was 0.53 (95% confidence interval [CI]: 0.49–0.57) and negative agreement was 0.70 (95% CI: 0.67 -0.72). For occupational doctors, positive agreement was 0.55 (95% CI: 0.38–0.69) and negative agreement was 0.71 (95% CI: 0.59–0.81). For internal tracer, positive agreement was 0.48 (95% CI: 0.42–0.53) and negative agreement was 0.68 (95% CI: 0.64–0.72). For general practitioners, positive agreement was 0.63 (95% CI: 0.53–0.72) and negative agreement was 0.51 (95% CI: 0.39- 0.62). For “other”, corresponding to “someone else” or “no one”., positive agreement was 0.56 (95% CI: 0.48–0.63) and negative agreement was 0.75 (95% CI: 0.71–0.79).

### Infection risk, by contact classification and tracer profile

Out of the 589 individuals included for our second research question, 375 (63.67%) were considered HRCs by the contact tracer, while the remainder were considered LRC. According to the guideline criteria, 243 (41.26%) were considered HRCs and the remainder LRCs. A total of 435 (73.85%) were immune at the time of contact. Out of all 589 individuals, 135 (22.92%) tested positive for COVID-19 shortly after contact. The secondary attack rate was 29.07% among HRCs, as defined by the contact tracer, and 12.15% among LRCs. The secondary attack rate was 27.16% among HRCs as defined by the guidelines and 19.94% among LRCs (Table 4).

**Table 4:** Results of PCR tests and risk ratio of infection according to type of contact classification (by the contact tracer or according to the guidelines) and the potential immunity of the individual

	Test		RR (95%CI)
	Positive	Negative	
Contact classification by CT			
HRC	109	266	3.1 (2.71–3.56)
LRC	26	188	1.0
Contact classification according to guidelines			
HRC	66	177	2.24 (1.94–2.60)
LRC	69	277	1.0
Potentially immune			
Yes	115	320	1.98 (1.55–2.53)
No	20	134	1.0

**Fig. 4:**
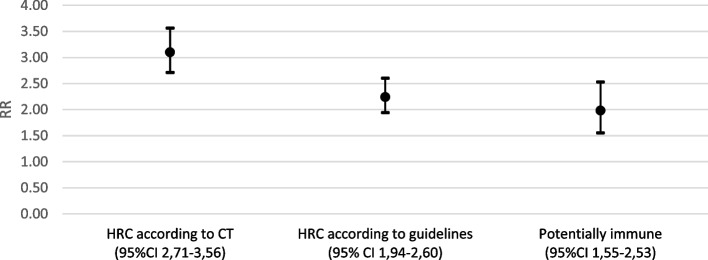
Interval plot of risk ratio of infection according to contact classification and immune status *p < 0.01

When a contact had been labelled a HRC by the contact tracer, the risk of infection was three times higher than the risk of infection for a LRC (RR: 3.1, 95% CI: 2.71–3.56). When a contact met the guideline-based HRC criteria, the RR was 2.24 (95% CI: 1.94–2.60) compared to a LRC. When an individual was potentially immune, as defined in the methods section above, the RR was 1.98 (95% CI: 1.98–2.53) compared to a non-immune individual (Fig. 4).

## Discussion

### Compliance with guidelines

We observed that, regardless of their profiles, contact tracers did not always rigorously follow contact tracing guidelines (positive agreement of 0.53 and negative agreement of 0.70). If the guidelines had been followed, we would have expected a PA and NA close to 1. There are several possible explanations for this lack of agreement:

Tracers may have deliberately not followed the guidelines after making their own assessment of the risk of infection. Indeed, it was observed during the pandemic that doctors might have been reluctant to apply externally imposed requirements concerning the provision and prioritization of patient care [9].

They may not have known the guideline criteria.

Participants may have erroneously reported the decision of the contact tracer and/or the guideline-based risk classification. We cannot verify this as self-reporting was the only data source used for this study.

Interviewed contacts may have deliberately offered selective information to the contact tracer as a result of mistrust or to obtain benefits associated with a particular contact classification. Mistrust is one of the obstacles to contact tracing described by Megnin-Viggars et al. [10] In their study, several obstacles to efficient contact tracing are identified at the level of the person being traced, not at the level of the contact tracer making their assessment, which is where the current study provides novel insights.

Respondents may have erroneously filled out the questionnaire, e.g. due to lack of attention or recollection bias.

Addressing these obstacles may improve the effectiveness and efficiency of contact tracing by making it more comprehensive and better targeted at individuals at highest risk of infection. Our study was observational, we did not find much literature on the actual implementation of contact tracing strategies. We therefore believe that our results can help to address the gap in knowledge on this topic.

### Comparison between CTs

Comparing the PA and NA results of the different contact tracer’s profiles reveals that they do not vary significantly from one profile to another. Our study does not, therefore, suggest that any of the profiles included applied the guideline criteria differently to the other profiles.

### Efficacy of contact classification

The secondary attack rate among contacts classified as HRCs by the contact tracers was 29%, which is higher than what we found in the literature [11–13] but comparable to the results of 27% found by Proesmans et al. who studied the performance of contact tracing in Belgium [14]. Our methodology made it possible to verify whether contact classification as high risk and low risk was useful, by comparing the infection risk in both groups. A Poisson regression found that the infection risk for HRCs was significantly higher than for LRCs. The RR between both groups was, at 3.1 for classifications by the contact tracer, which was significantly greater than for guideline-based classifications, for which the RR was 2.2. Contact tracers may thus have applied criteria that are not included in the definition of a HRC to assess the contact’s risk of infection. They also may have had access to additional information for processing their case (existence of a cluster in the company, access to the quantitative results of the index case’s PCR test, which may have suggested high viral shedding [15], etc.). We did not come across any research that used the same methodology as ours to calculate the RR of infection between HRCs and LRCs. Several studies, however, have applied a similar methodology to compare positivity rates among HRCs and LRCs. Sahoo et al. [16], Velhal D et al. [17] and Sharma et al. [18] collected information about 3411, 1486 and 1430 health care workers respectively. They were classified as HRCs and LRCs. Sahoo et al. obtained positivity rates of 3.8% for HRCs and 1.9% for LRCs. Velhal D et al. obtained positivity rates of 9.01% for HRCs and 2.72% for LRCs. Sharma et al. obtained positivity rates of 19.5% for HRCs and 0.6% for LRCs. We observe that their contact classification was similar and based on the CDC criteria. Risk stratification in contact tracing was found to be effective, however as it was in our study, even though the risk ratio was not calculated in these studies. Their positivity rates are lower than ours which could be attributed to the timing of the data collection (less contagious variants) and the limitations of our study. While they focused on populations of healthcare workers, we opted to include other professional fields.

The third variable, potential immunization, was included because we suspected that it would reduce transmission risk, as has been described [19, 20], which could have biased our results. Surprisingly, the Poisson regression showed the opposite, with an RR of 2.0 for potentially immune participants compared to non-immune participants. Numerous factors could explain this inconsistency. Our criteria for potential immunization were broad and probably resulted in the inclusion of non-immune participants. Furthermore, we cannot be certain that participants considered to be “non-immune” were indeed not immune, as case under-ascertainment was common during the first waves of COVID-19 infections [21]. Potentially immune healthcare workers may have been assigned to COVID-19 units, thus being at higher risk of infection but also were more often vaccinated and more intensely screened than other respondents were. Potentially immunized workers may have taken more risk in relation to exposure to others as a consequence of feeling protected from infection and severe disease [22]. The emergence of the Delta variant may have partially neutralized the protection offered by the vaccine against infection [23].

We specifically evaluated the risk classification of contacts and found it to be useful for identifying individuals at high risk of infection. We also demonstrated that targeting of testing is improved when a knowledgeable tracer performs the risk assessment.

### Limitations

Our study has several limitations. It was retrospective and based on an anonymous online survey, both factors which may have reduced the accuracy of the data. Participants could stop filling out the questionnaire at any time, which may also have reduced accuracy and completeness. Although participants received information describing the subject of the study in their email, this information may have led to a selection bias.

Healthcare workers were overrepresented in the study population. This is unsurprising because the organization through which participants were recruited (CESI) is particularly active in this sector, and healthcare workers were probably more exposed to COVID-19, leading to more COVID-19 contacts at work [24]. Stratification by job category would have been useful as risk differs significantly from one field to another. It was unfortunately not feasible due to the sample size.

The questionnaire was created during the first half of 2021, shortly after the start of vaccination and before the administration of booster doses. For this reason, we defined vaccination status rather broadly.

Furthermore, in the section concerning compliance, results for individuals traced by “someone else” or “no one” should be interpreted with caution, as many individuals in this category answered “I don’t know” to the question about their contact classification. This latter answer was an exclusion criterion. There was therefore a selection bias that may have influenced the PA and NA.

Finally, the respondents knew the results of their test following contact, which may have had an impact on their questionnaire answers. For example, participants who tested positive may have seen the risk as greater when they replied to the questionnaire than when they responded to the contact tracer. This may have had an impact on both the calculation of the PA/NA and the comparison of the infection risk of HRC and LRC, as per contact tracer and guideline-based classification.

## Conclusion

In this study, we evaluated the implementation of contact tracing in the work environment. We investigated the gap between guideline-based contact classification into high risk and low risk, and contact classification by contact tracers. We also evaluated whether contact tracers in general, and different profiles of contact tracers in particular, were better able to target scarce testing resources to individuals at highest risk of infection. Contamination in workplaces was specifically assessed in Belgium. There was a specific interest, therefore, in examining contact tracing strategies in this specific setting. This is, to the best of our knowledge, the first study to assess contact tracing in a work environment, the implementation of risk classification criteria by contact tracers in practice, and the ability of contact tracers with different profiles to fulfill their task of stratifying contacts according to their risk of infection.

Our study indicates that contact tracers do not always rigorously apply contact tracing guidelines, as set out by public health authorities. While they label more individuals as HRCs, their secondary attack rate remains higher than what it would be if it was based on strict implementation of the guideline requirements. This shows that a knowledgeable tracer can target testing better than a general guideline can. The risk of infection was higher among HRCs than among LRC for both types of classifications (tracer and guideline criteria), which confirms that both types of assessment are valid, without discounting the higher efficiency of the tracer’s assessment.

### Supplementary Information

**Additional file 1. **

## Data Availability

Data may be made available upon reasonable request to the corresponding author.

## Abbreviations

COVID-19: Coronavirus Disease 2019
GP: General Practitioner
HRC: High Risk Contact
LRC: Low Risk Contact
NA: Negative Agreement
PA: Positive Agreement
RR: Risk Ratio
SARS-CoV-2: Severe Acute Respiratory Syndrome Coronavirus 2
