# Supplementary material for: COVID-19 contact tracing at work in Belgium - how tracers tweak guidelines for the better
Source: BMC Public Health. 2023 Nov 3;23:2148. doi: 10.1186/s12889-023-16911-1 (PMC10623756; doi:10.1186/s12889-023-16911-1)
Supplement: Supplementary file 1 — Additional file 1. [file 12889_2023_16911_MOESM1_ESM.docx]

**Appendix 1: Questionnaire**

**Q0: In which language do you wish to complete this questionnaire?**

- French
- Dutch

**Q1: What is your age?**

_______

**Q2: What is your gender? (please select the option that corresponds to you)**

- Male
- Female
- Other
- I prefer not to answer

**Q3: What is the highest educational level you have reached?**

- No educational qualifications
- Primary school leaving certificate
- Secondary school leaving certificate (general)
- Secondary school leaving certificate (vocational)
- Higher education degree
- I prefer not to answer

**Q4:** **Which statement corresponds best to your work activity?**

- Teaching
- General health care (work in hospital, primary care, care home etc.)
- Social work
- Other

**Q4bis : Are you willing to let us know your occupation?**

________________

**Q5: Since the start of the pandemic, have you been in contact with a colleague infected with the coronavirus while at work?**

**By contact we mean that you have been with this person when they were contagious (during the two days before they became ill or the two days before they tested positive for the coronavirus).**

- Yes
- Not as far as I know

**Q6: In which month did this contact take place? If you do not remember the exact date, please indicate the first day of the month (example: 01/10/2020).**

________________

**Q7 : Have you been vaccinated against the coronavirus?**

- No
- Yes, 1 dose
- Yes, 2 doses

**Q7 bis: When were you vaccinated? (date of last dose received) If you can no longer remember the exact date, please state the first day of the month (example: 01/04/2021).**

________________

**Q8: Have you tested positive for the coronavirus?**

- No
- Yes

**Q8 bis: When did you test positive? (If you do not remember the exact date, please indicate the first day of the month, example: 01/10/2020)**

________________

**Q9: Have you been officially informed that you were in contact with a positive case?**

- Yes
- No

**Q10: Who informed you of this contact? (you can choose more than one answer)**

- The colleague who was infected
- A person at work (my employer, human resources, the prevention officer etc.)
- Your (CESI) occupational doctor.
- Other

**Q11: Was your contact with your colleague assessed as a low-risk contact or a high-risk contact?**

- Low-risk contact
- High-risk contact
- I don’t know

**Q12: Who made this assessment?**

- The CESI occupational doctor
- Someone from my company (employer, HR, prevention adviser)
- My general practitioner
- Someone else
- No-one

**Q13: Were you placed in quarantine following this contact?**

- Yes
- No

**Q13bis: Did you develop symptoms of COVID following this contact?**

- Yes
- No

**Q14: Did you take a test (PCR test) following this contact?**

- Yes
- No

**Q14 bis: Was this test positive?**

- Yes
- No

**Q15: Were you in contact with the infected colleague for at least 15 minutes in total?**

- Yes
- No

**Q15 bis: During this contact, were you at a distance of less than 1.5m?**

- Yes
- No

**Q15 ter: Also during this contact, were you face to face (for example having a conversation), with either person not wearing a mask correctly (nose and mouth completely covered)?**

- Yes
- No

**Q16: Were you in direct physical contact with the infected colleague? (handshake, hug etc.)**

- Yes
- No

**Q17: Were you identified as a close contact by the “Coronalert” app?**

- Yes
- No
- I do not use the app

**Q18: Were you in a vehicle for 15 minutes with the infected colleague (car, public transport etc.)?**

- Yes
- No

**Q19: Did you eat/drink with the infected colleague (coffee break, meal etc.)?**

- Yes
- No

**Q20: Did you share equipment with the infected colleague (for example: ballpoint pen, file, keyboard, mouse, photocopier etc.)?**

- Yes
- No
- I don’t know

**Q20bis: Were both hands and the equipment disinfected when it was shared?**

| Never | Sometimes | Often | Systematically |
| --- | --- | --- | --- |

**Q21: Did you and the infected colleague share the same workstation? (using the same computer or the same equipment at different times)**

- Yes
- No
- I don’t know

**Q22: Were you and the infected colleague considered as being in a “work bubble”?**

**Work bubble: some companies put employees in pairs (or sometimes larger groups) to avoid mixing teams and limit transmission of the virus within the company.**

- Yes
- No
- I don’t know

**Q23: Was there a cluster (or focus of infection) at your work?**

**A cluster means several cases (at least two) of COVID in the same workplace with suspected infection having occurred between the individual persons**

- Yes
- No
- I don’t know

**Q24: If you have not found the type of contact that resulted in your infection in the questions above, please tell us in a few words the time/situation when you think you were infected?**

____________________________________

**Q25: How do you usually comply with infection control measures at work?**

- **social distancing:**

| Never | Sometimes | Often | Systematically |
| --- | --- | --- | --- |

- **wearing a mask:**

| Never | Sometimes | Often | Systematically |
| --- | --- | --- | --- |

- **hand hygiene:**

| Never | Sometimes | Often | Systematically |
| --- | --- | --- | --- |

**Q26: In this specific contact situation, do you think you complied with the infection control measures?**

| Not at all | Not very well | Appropriately | Perfectly |
| --- | --- | --- | --- |
